# Supplementary material for: Uptake and perceptions of oral HIV self-testing delivered by village health teams among men in Central Uganda: A concurrent parallel mixed methods analysis
Source: PLOS Glob Public Health. 2023 Jun 14;3(6):e0002019. doi: 10.1371/journal.pgph.0002019 (PMC10266653; doi:10.1371/journal.pgph.0002019)
Supplement: Attachment — Submitted filename: Response_to_Reviewers.doc [file pgph.0002019.s001.doc]

**RESPONSE TO REVIEWERS’ COMMENTS**

**Reviewer #1:**

Thank you for inviting me to review "Uptake and perceptions of oral HIV self-testing delivered by Village Health Teams among men in Central Uganda: A concurrent parallel mixed methods analysis"

**Comments # 1: In Abstract**

1) Methods. Line number 35: What types of analysis were used for quantitative data? Please mention it. In this section, the authors only highlighted qualitative data analysis.

We used descriptive statistics for the quantitative data. See line 35.

2) Results. Who were your significant others? Was there any association? Please highlight these.

The significant others were friends and family. We have clarified that. See line 42 and 43.

**Comments # 2: Introduction**

3) The author should include some references that have summarized HIV status awareness across the globe.

We have included the global prevalence of HIV testing (85%). See line 53 and 54.

4) The author also ought to include some studies that have been conducted not only in developing countries but also in developed countries to support this manuscript.

We have included references of systematic reviews summarizing studies from the high-, middle- and low- resource settings. See line 66.

5) The introduction part highlighted only Uganda and Sub-Saharan African-related studies.

We have included citations of work done elsewhere in the world. See line 66.

**Comments # 3: Methods**

**Study design and setting.**

6) What were the reasons for conducting this study only for men except for women?

We opted to enroll only men because they lag behind in HIV testing and HIV status awareness compared to women. We included this in the first paragraph of the introduction section. See line 54-64.

7) Was any previous study available among women in this region?

Yes. In Uganda, prior research on HIVST was conducted among key populations which including female sex workers, fisher folk and their partners, injecting drug users and male partners of pregnant women. See line 67-68.

8) How many Village Health Teams (VHTs) were directly involved to conduct this survey? Please mention.

A total of 30 VHTs were involved in the study. See line 123-124.

**Study participants.**

9) Why the study was selected ≥ 15 years age of the study participants? ≤ 15 years age of the participants were safe from HIV or the prevalence rate was too little in this region or no statistics were available?

Our primary population of interest were adults aged ≥ 18 years however, we extended the inclusion limit 15years for emancipated minors since they can independently test and receive HIV results, and consent to participate in research. See line 99-101.

10) Line number 107-108: This is unnecessary. Please omit this line.

We have deleted the repetition of the study aim. See line 106.

**Sampling and sample size estimation.**

11) Please highlights the sample size estimation formula. Line number 117: How many participants were included in the qualitative study? Please clear it.

We have included the formula. See line 110-114. In the qualitative study, the sample size was based on data saturation (line 121), which was ultimately 14 men as indicated in the results section. See line 189-191.

**Study procedures.**

12) Which type of questionnaires were used in the in-depth interview? Was it structured/unstructured/ semi-structured?

We used topic guides comprising of open-ended questions. See line 150.

**Data analysis.**

13) The data were only generalized by percentage. Please revise the analysis and perform a higher level of analysis to find out the significant association between different variables.

In this analysis, we aimed to assess uptake and perceptions of oral HIVST delivered by Village Health Teams. We assessed uptake quantitatively using descriptive statistics (see line 161-162), and perceptions using qualitative analyses. We therefore did not assess for associations between variables because it was not part of the aim present in this paper.

**Comments # 4: Results**

14) Line number 180: Please use the numerical value 25 instead of twenty-five participants.

We have added the numerical value (25). We had opted to write it in words because it was beginning a sentence. See line 185.

15) Table 1: Please correct the spelling of Moslem.

We have corrected the spelling to Muslim. See Table 1, line 197.

16) Table 2: Please revise the variables tested =>12 months ago.

We have revised the variable to Tested more than 12 months. See Table 2 and 3, line 234.

17) Line number 239: Please use the numerical value 9 instead of nine participants.

We have added the numerical value (9). We had opted to write it in words because it was beginning a sentence. See line 247.

18) Line number 247: Please use the numerical value 46% in place of forty-six percent.

We have added the numerical value (46%). We had opted to write it in words because it was beginning a sentence. See line 256.

19) Table 3: Please mention the AOR in the rows of Self-reported HIVST uptake (Yes), n (%; 95% CI), and HIVST uptake verified by viewing the used kit (Yes), n (%; 95%CI).

We did not report the AOR because the analyses in this paper did not include assessing for associations between HIVST uptake and the independent variables.

**Comments # 5: Discussions**

1) The discussion sections should be revised. Line number 400: Please highlight the elaboration of PWH before using the abbreviations.

We reviewed the discussion section and corrected any unclear sentences and grammatical errors. We have also replaced the abbreviation PWH with PLHIV, and defined it in full at first appearance. See line 53.

**Comments # 6: References**

1) Some references were used in this manuscript which is more than 10 years. Please updated the references including 20, 30, 37, 41, 49, 58, and 66.

Yes, we reviewed our list of references and found some that were over 10 years old. However, most of these had informed our research and were the best-fit justification evidence we found. The references included: 1) frameworks by Aday and Andersen from 1974 which guided our understanding of concepts in access to healthcare services; 2) sample size calculation formula by Bennet et al in 1991; 3) reference prevalence studies at that time which informed our sample size calculation; 4) reference HIVST studies in Sub-Saharan Africa by Choko et al in 2011 and 2015 which informed the study design and procedures among others. We have also updated our references and have included recent studies. See line 66.

**Overall comments**

There are some grammatical errors as well as improving the English language. It is noticed that the structure of many sentences is misleading. Please revise the manuscript correctly, avoiding grammatical errors.

We have reviewed the manuscript and corrected any unclear sentences and grammatical errors.

**Reviewer #2:**

It's a pertinent study to understand the uptake of HIV self-testing kits and the perception of this service delivered through a local team like village health team in Mpigi district in Uganda.

It has the potential to inform implementation of programs that are community based for better uptake of HIV testing.

The research question, objectives, methods are all-coherent and are explained in depth.

Results are well structured and laid out. A few minor clarifications to be given by the authors in the methods –

1) Rationale behind choosing 10 days as duration after which follow up was done to see if the test was utilised can be explained.

We have included this in the study procedures on page 7, line 139-142 stating that “Since HIVST was relatively a new testing approach, offering 10 days for HIVST was considered sufficient time for each participant to reflect and decide to test or not, comparable to studies on community-based unsupervised oral HIVST held elsewhere in SSA”.

2) Whether experiences of the village health teams was considered in order to comment of feasibility of such a programme including the challenges? If not maybe mention this as a limitation or further area to be explored before scaling this up.

Yes, we captured the experiences of village health teams via in-depth qualitative interviews at the end of the follow-up period. These findings will be reported in a separate publication so their details were not included here.

3) Marital status was asked to all men - were only heterosexual men included in the study? Was sexual orientation asked about? if so please specify if there were any implicit assumptions related to this and state under methods/limitations accordingly.

Yes, all participants were asked for their marital status. Since our study was in the general population, we focused on cis-men identifying with the male gender so did not make in-depth inquiry on sexual orientation. We have included this in the limitations of the study. See page 25, line 463-466.

4) There is a chance that the choice to purposely interview people who utilized the test kit and those who volunteered among them to share their experience, would have influenced the perceptions shared instead of exploring both those who utilized and did not utilize the kit. Which might have given a better understanding. This is something to consider for the limitation section or explain the rationale behind this criteria.

In this paper, we focused on men that used HIVST so have acknowledged this limitation. See page 25, line 466-469. However, as part of the larger study, we interviewed some of the men that did not use oral HIVST and intend to write and publish it separately.
